# Association between estimated glomerular filtration rate slope and cardiovascular disease among individuals with and without diabetes: a prospective cohort study

**DOI:** 10.1186/s12933-023-02008-x

**Published:** 2023-10-04

**Authors:** Azra Ramezankhani, Fereidoun Azizi, Farzad Hadaegh

**Affiliations:** 1 Prevention of Metabolic Disorders Research Center, Research Institute for Endocrine Sciences, Shahid Beheshti University of Medical Sciences, Tehran, Iran; 2 Endocrine Research Center, Research Institute for Endocrine Sciences, Shahid Beheshti University of Medical Sciences, Tehran, Iran

**Keywords:** Glomerular filtration rate, Cardiovascular disease, Slop, Diabetes

## Abstract

**Background:**

Previous studies have reported an association between a significant decline in estimated glomerular filtration rate (eGFR) over time and an increased risk of cardiovascular disease (CVD). This study aimed to investigate the association between the eGFR slope and CVD among individuals with and without diabetes.

**Methods:**

This prospective cohort study was conducted within the Tehran Lipid and Glucose Study (TLGS) framework. We studied 6919 adults aged 20–70 years, including 985 with diabetes and 5934 without diabetes. The eGFR slope was determined based on repeated measurements of eGFR through linear mixed-effects models. A multivariable Cox proportional hazard model was employed to evaluate the association between eGFR slope, both in continuous and categorical form, and the risk of CVD.

**Results:**

The slopes of eGFR exhibited a bell-shaped distribution, with a mean (standard deviation (SD)) of -0.63 (0.13) and − 0.70 (0.14) ml/min per 1.73 m^2^ per year in individuals with and without diabetes, respectively. During a median follow-up of 8.22 years, following the 9-year eGFR slope ascertainment period, a total of 551 CVD events (195 in patients with diabetes) were observed. Among individuals with diabetes, a steeper decline in eGFR slope was significantly associated with a higher risk of CVD events, even after adjusting for baseline eGFR, demographic factors, and traditional risk factors for CVD; slopes of (-1.05 to -0.74) and (-0.60 to -0.52) were associated with 2.12 and %64 higher risks for CVD, respectively, compared with a slope of (-0.51 to 0.16). Among individuals without diabetes, the annual eGFR slope did not show a significant association with the risk of CVD.

**Conclusion:**

Monitoring the eGFR slope may serve as a potential predictor of CVD risk in individuals with diabetes.

**Supplementary Information:**

The online version contains supplementary material available at 10.1186/s12933-023-02008-x.

## Background

Cardiovascular disease (CVD) is a serious health issue and remains the primary cause of disability and mortality worldwide [1]. An estimated 18.5 million people died from CVDs in 2019, representing 32% of all global deaths [2]. In the Middle East and North Africa (MENA) countries, CVDs are responsible for approximately 45% of all deaths [2]. According to a study in 2019, CVD was the leading cause of mortality in Iran, accounting for approximately 46% of all deaths [3]. Further, it was found that at age 20, the remaining lifetime risk for CVD was 66.7% and 52.0% in Iranian men and women, respectively [4, 5].

It is well established that chronic kidney disease (CKD), diagnosed by an estimated glomerular filtration rate (eGFR) below 60 mL/min/1.73 m^2^ is an important risk factor for CVD and cardiovascular mortality [6]. CKD is a long-term condition characterized by a gradual and progressive loss of kidney function over time and can lead to end-stage renal disease (ESRD), the final permanent stage of CKD, if left untreated [7]. Therefore, accurately assessing the level of GFR and its magnitude of change over time is critical to detecting CKD, understanding its severity, and making decisions about diagnosis, prognosis, and treatment [8]. Previous meta-analyses have demonstrated that 30% and 40% declines in eGFR were associated with subsequent risks of ESRD and mortality in individuals with and without diabetes. However, these studies calculated the percentage of change using only two measurements and did not account for the trajectory of eGFR over time [9, 10].

In recent years, there has been a growing interest in the assessment of the slope of eGFR over multiple time points to determine individuals at high risk for the development of ESRD or CVD, particularly in clinical trials [11, 12]. Using data from the ADVANCE-ON study, an annual substantial decrease in eGFR (eGFR slope <-1.63 mL/min/1.73 m^2^ per year) among individuals with diabetes was significantly associated with the subsequent risk of CVD, compared with a stable change in eGFR (-1.63 to 0.33 mL/min/1.73 m^2^ per year) [13]. Another study found that among individuals with CKD, having a slope of one standard deviation (SD) below the average was associated with a 19% higher risk of CVD [11].

To our knowledge, no study has investigated the correlation between eGFR slope and subsequent incident cardiovascular events in the MENA region, with a high prevalence of diabetes, hypertension, and metabolic syndrome [14, 15], factors are known to affect both kidney function [16, 17] and cardiovascular health [18, 19].

Therefore, this study utilized data from adult individuals participating in the Tehran lipid and Glucose Study (TLGS) cohort with the following objectives: (1) to estimate the eGFR slope over 12 years, and (2) to determine the association between eGFR slopes and CVD events, separately among individuals with and without diabetes.

## Methods

### Study population

**Fig. 1:**
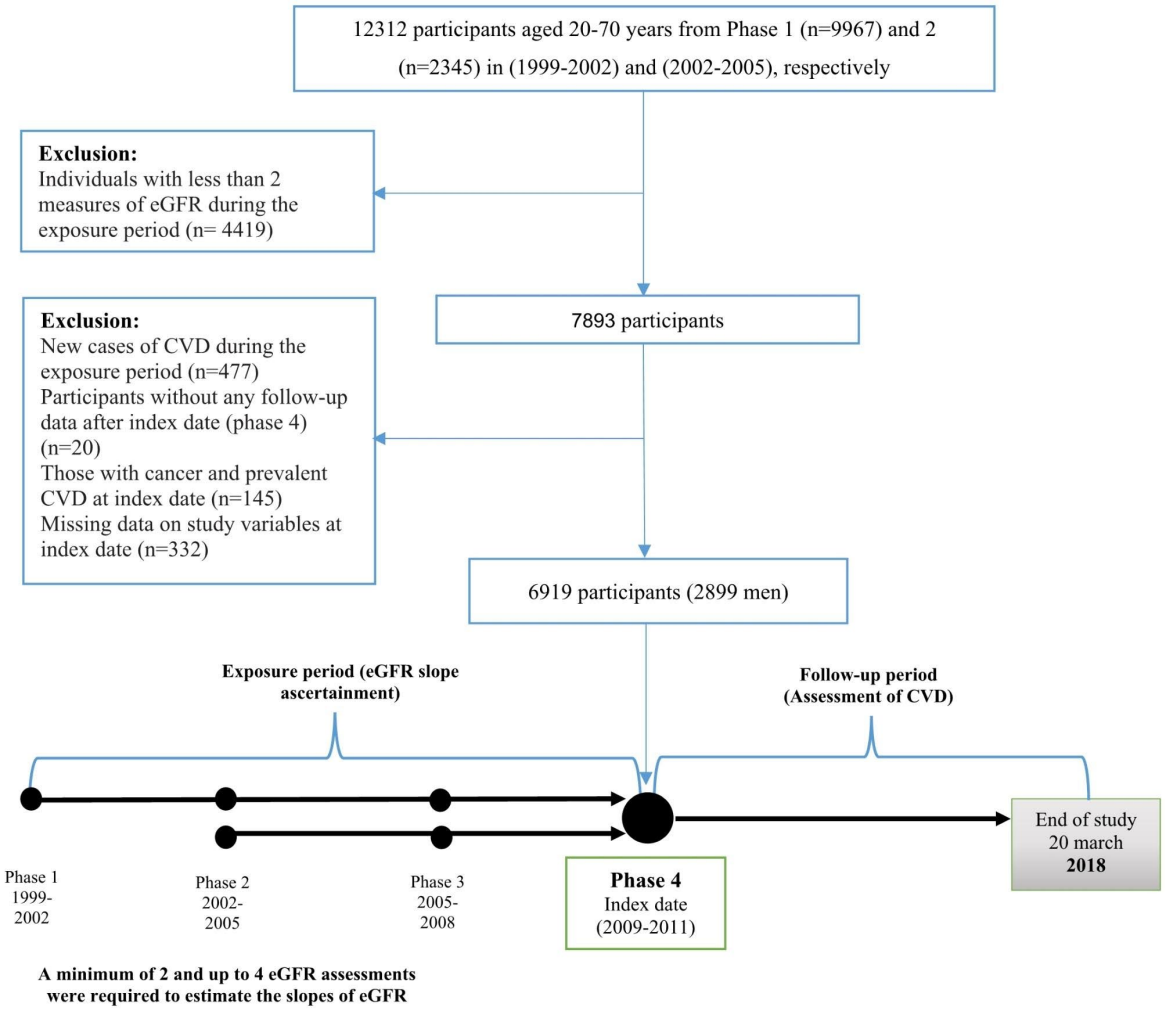
Flowchart of sample selection for the study. **CVD**: cardiovascular disease; **eGFR**: estimated glomerular filtration rate

The TLGS is a long-term (20-year) ongoing population-based cohort study conducted in Tehran, the capital of Iran. It aims to investigate the risk factors and outcomes related to non-communicable diseases. A detailed description of the design and methods of the TLGS study has been published elsewhere [20, 21]. In brief, a total of 15,005 individuals aged ≥ 3 years were recruited in phase 1 (1999–2002), and 3550 new subjects were added in phase 2 (2002–2005). Relevant data at the entrance were collected using the questionnaire interviews and health examinations, and measurements were repeated triennially in phase 2, phase 3 (2005–2008), phase 4 (2009–2011), phase 5 (2012–2015), and phase 6 (2015–2018). In this study, the 20 years was divided into two sub-periods: the “exposure period” which spanned from the entrance into the study until phase 4, and the “follow-up period” which encompassed the time from phase 4 until the end of the study. Therefore, phase 4 was defined as the index date (baseline). We selected 12,312 participants aged 20–70 years from phase 1 (n = 9967) and phase 2 (n = 2345) and excluded those individuals (n = 4419) with less than two measures of eGFR and new cases of CVD (n = 477) during the exposure period. Of the remaining participants, we excluded those without any follow-up data during the study period (n = 20), those with cancer and prevalent CVD (n = 145), and those with missing data on study variables (n = 332) at the index date. Finally, a total of 6919 (2899 men) remained as the study participants and were followed until the end of the study (20 March 2018) (Fig. 1).

Study protocols were approved by the ethical committee of the Research Institute for Endocrine Sciences of Shahid Beheshti University of Medical Sciences, Tehran, Iran, and written informed consent was obtained from each study participant.

### Measurements

Information on age, education, marital status, smoking status, medication use, and family history of CVD (FH-CVD) was obtained using standardized questionnaires at each phase. Height and weight were measured using the standard protocol, and body mass index (BMI) was calculated as weight (kilograms) divided by height (meters) squared (kg/m^2^). Blood pressure was measured by trained technicians using a standard mercury column sphygmomanometer. The systolic and diastolic blood pressure (SBP and DBP) values were then calculated as the average of two measurements. Blood samples were collected for fasting plasma glucose (FPG), 2-h post-load plasma glucose (2-hPG), total cholesterol (TC), triglycerides (TG), high-density lipoprotein cholesterol (HDL-C), and creatinine levels. The history of pan-retinal photocoagulation (PRP), which was carried out during the exposure period was considered as a surrogate for incident severe non-proliferative and proliferative diabetic retinopathy (NPDR/PDR) [22]. GFR was estimated by use of the Chronic Kidney Disease Epidemiology Collaboration (CKD-EPI) equation as follows:

eGFR = 141× min (Scr/K, 1)^a^ × max (Scr/ K, 1)^−1.209^ × 0.993^age^ ×1.018 [if female] ×1.159 [if black].

“Scr” is serum creatinine that was measured in mg/dl, “K” is 0.7 for women and 0.9 for men, “a” is 0.329 for women and − 0.411 for men, “min” indicates the minimum of Scr/K or 1, and “max” indicates the maximum of Scr/K or 1. Before calculation, we multiplied creatinine values by 0.95 to obtain standardized SCr [23, 24].

### Definition of terms

Educational level was classified into three categories: < 6 years, 6–12 years, and > 12 years of schooling. We categorized marital status into three categories: single, married, and widowed/divorced. The classification of smoking status involved three categories: current smoker, past smoker, and never smoker. A current smoker was described as an individual who smokes cigarettes or other smoking implements daily or occasionally. Never-smokers were defined as subjects who had never smoked, while past smokers were defined as individuals who had stopped smoking for at least one year before the start of the study. FH-CVD was defined as the presence of a female first-degree relative younger than 65 years, or any male first-degree relative younger than 55 years, with a history of CVD events. Hypertension was defined as a blood pressure level ≥ 130/80 mmHg or the use of antihypertensive medication [25]. Type 2 diabetes mellitus (T2DM) was defined as FPG > 7 mmol/L or 2-hPG > 11.1 mmol/L or treatment with antidiabetic drugs. Hypercholesterolemia was defined as TC ≥ 5.17 mmol/L (200 mg/dl) or the use of lipid-lowering drugs. Renin-angiotensin-aldosterone system inhibitors (RAAS-I) included angiotensin-converting enzyme inhibitors (ACEi) and angiotensin receptor blockers (ARBs).

### Primary exposure

The eGFR slope was the primary exposure which is a measure of the rate of change in eGFR over time and estimated using eGFR values collected from the beginning of the study (phase 1 or 2) until phase 4. Each participant had at least two eGFR measurements, including baseline and index date, which were necessary to estimate the slope of eGFR. Some participants may have had up to four eGFR measurements.

### Primary outcomes

Time to first CVD event was the primary outcome, which was ascertained from the index date through late 2018. Participants were censored for non-CVD death, withdrawal from the study, or reaching the end of the study without experiencing any CVD events.

Details of the collection of CVD events have been published previously [26]. In brief, all TLGS study participants were followed up annually for any medical events from the baseline examination until the date of the first documented events. An expert panel reviewed the data to ensure the accuracy of them. We defined CVD as definite myocardial infarction (MI) (diagnosed by electrocardiogram (ECG) and biomarkers), probable MI (positive ECG finding, cardiac symptoms, or signs plus missing biomarkers or positive ECG findings plus equivocal biomarkers), unstable angina (new cardiac symptoms or changing symptom patterns and positive ECG findings with normal biomarkers), angiographic proven coronary heart disease (CHD), congestive heart failure (CHF), stroke and death attributable to CVD events.

### Statistical methods

#### Estimation of slopes

We used an unadjusted linear mixed-effects model (LMEM) of longitudinal eGFR to estimate the slopes of eGFR separately in men (n = 2899) and women (n = 4020). The LMEM incorporates both fixed and random effects. Fixed effects represent the average change over time, and random effects capture the individual variations around the fixed effects [27, 28]. We incorporated both a random intercept and random-slope component in LMEM with a general correlation structure of the random effects for the residuals based on a series of likelihood ratio tests. After estimating slopes for each person, the pooled data set of 6919 individuals was divided into two subsets of diabetes (n = 985) and non-diabetes dataset (n = 5934) for subsequent analyses. Among the total population, 669 individuals (9.6%) had two measures of eGFR, 2351 individuals (34%) had three measures, and 3899 individuals (56.4%) had four measures of eGFR. The supplementary Table 1 displays the number of eGFR values categorized by sex, diabetes status, and quintile of slopes. Approximately 90% of individuals, both with and without diabetes, had a minimum of three eGFR measurements, while over 55% had a minimum of four eGFR measurements during the exposure period.

#### Descriptive analysis

Summary statistics for population characteristics at both entrances into the study and index date were computed overall and by diabetes status. Mean (standard deviation (SD)) and number (percent change) were used for continuous and categorical variables, respectively. For continuous variables with skewed distribution such as TG, FPG, and TC, the median (interquartile range (IQR)) is displayed.

The crude incidence rate and 95% confidence interval (95% CI) per 1000 person-years for CVD were calculated by diabetes status and across quintiles of eGFR slopes.

Baseline characteristics were also compared across quintiles of eGFR slopes in subjects with and without diabetes. We further compared baseline characteristics between participants and non-participants. Non-participants included subjects with less than two measures of eGFR during the exposure period, individuals without any follow-up data, and those with missing data on study variables at the index date. Differences were assessed by analysis of variance (ANOVA), Kruskal-Wallis test, t-test, and Mann-Whitney U test, as appropriate.

#### Cox models

We performed the Cox proportional hazards model to estimate the association between eGFR slope and incident CVD stratified by diabetes status. The eGFR slopes were included in models as continuous (mL/min/1.73 m^2^ per year) and categorical (quintile) forms. In categorical form, the fifth quintile was defined as the reference category. No significant effect modification by gender was observed for eGFR slopes among those with and without diabetes (P for interaction > 0.05). Therefore, the analysis was conducted without stratifying by gender.

Model 1 was adjusted for age and gender. Model 2 was further adjusted for BMI, smoking, hypertension, FPG, hypercholesterolemia, FH-CVD, marital status, and educational level. For individuals with diabetes, model 2 was further adjusted for glucose-lowering drugs. Model 3 was adjusted for variables in model 2, as well as eGFR at index date and RAAS-I drugs. In individuals with diabetes, model 3 was further adjusted for NPDR/PDR.

The linear trend across quintiles was tested by including quintiles as a single continuous variable in Cox models.

We also performed a multivariable Cox regression, in which the primary exposure was the slope of eGFR, modeled with restricted cubic splines (knots at 5th, 25th, 50th, 75th, and 95th percentiles) to evaluate the non-linearity of the associations.

Statistical analysis was performed using R (version 4.2.1), and statistical package for the social sciences (SPSS) version 20. The statistical significance level was set at a two-sided P value < 0.05.

## Results

### Description of the population

Total participants had a mean (SD) age of 39.5 (12.6) years at the time of study enrollment, and 58.1% were women (Supplementary Table 2). The eGFR slopes were estimated for all participants over the exposure period, with a median of 9 years (range: 3–12 years) from study enrollment until the index date. The mean (SD) eGFR slope was − 0.63 (0.13) and − 0.70 (0.14) mL/min/1.73 m^2^ per year for individuals with and without diabetes, respectively (P < 0.001) (Supplementary Table 3). The distribution of annual eGFR slopes in the study population is shown by diabetes status in Supplementary Fig. 1. Also, distributions are shown across quintiles of eGFR slopes separately among individuals with and without diabetes in Supplementary Figs. 2 and 3, respectively.

During a median follow-up time of 8.22 years, a total of 551 CVD events were observed in the overall population, with 195 occurring in individuals with diabetes (median follow-up time of 8.34 years), and 356 occurring in those without diabetes (median follow-up time of 8.22 years).

The crude incidence rates (95% CI) were 27.6 (24.0-31.8) and 7.6 (6.9–8.5) per 1000 person-years among those with and without diabetes, respectively.

Significant differences were observed in most characteristics, both at the time of study enrollment and the index date, between individuals with and without diabetes. (Supplementary Tables 2 and 3).

Tables 1 and 2 represent characteristics of participants with and without diabetes, respectively, across quintiles of eGFR slopes at index date. In individuals with diabetes (Table 1), statistically significant differences in all baseline characteristics (except for DBP and FH-CVD) were observed across quintiles of the eGFR slope. A decreasing pattern was observed for BMI as individuals moved from quintile 1 to quintile 5 of the eGFR slope. However, there was a consistent trend of increasing values for SBP, TG, prevalence of hypertension, and use of RAAS-I drugs (all P values for trend < 0.001).

**Table 1:** Characteristics of participants with diabetes (n = 985) according to quintiles of eGFR slopes at index date*

Characteristics	Quintile 1 (n = 197)	Quintile 2 (n = 197)	Quintile 3 (n = 197)	Quintile 4 (n = 197)	Quintile 5 (n = 197)	P Value	P trend
Slope range (ml/min per 1.73 m^2^ per year)	(-1.06, -0.75)	(-0.75, -0.67)	(-0.67, -0.61)	(-0.61, -0.52)	(-0.52, -0.17)		
Slop, mean (SD)	-0.82 (0.06)	-0.70 (0.02)	-0.63 (0.02)	-0.56 (0.02)	-0.43 (0.07)	< 0.001	< 0.001
Age, mean (SD)	37.7 (9.1)	44.3 (8.8)	48.9 (9.2)	53.1 (8.8)	57.2 (8.1)	< 0.001	< 0.001
Female sex, n (%)	130 (66.0)	147 (74.6)	144 (73.1)	118 (59.9)	58 (29.4)	< 0.001	< 0.001
Educational level, n (%)							
< 6 years	48 (24.4)	79 (40.1)	107 (54.3)	114 (57.9)	110 (55.8)	< 0.001	< 0.001
6–12 years	117 (59.4)	102 (51.8)	72 (36.5)	70 (35.5)	65 (33.0)		< 0.001
> 12 years	32 (16.2)	16 (8.1)	18 (9.1)	13 (6.6)	22 (11.2)		0.089
Marital status, n (%)							
Single	4 (2.0)	4 (2.0)	4 (2.0)	3 (1.5)	0 (0.0)	0.001	0.105
Married	178 (90.4)	167 (84.8)	150 (76.1)	148 (75.1)	165 (83.8)		0.009
Widowed/Divorced	15 (7.6)	26 (13.2)	43 (21.8)	46 (23.4)	32 (16.2)		0.001
Smoking status, n (%)							
Never smoker	150 (76.1)	156 (79.2)	162 (82.2)	150 (76.1)	137 (69.5)	0.001	0.089
Past smoker	15 (7.6)	10 (5.1)	12 (6.1)	26 (13.2)	33 (16.8)		< 0.001
Current smoker	32 (16.2)	31 (15.7)	23 (11.7)	21 (10.7)	27 (13.7)		0.189
BMI (kg/m^2^), mean (SD)	30.9 (6.1)	30.2 (5.3)	31.2 (5.6)	29.8 (4.7)	28.5 (4.4)	< 0.001	< 0.001
SBP (mm Hg), mean (SD)	120.1 (18.6)	126.5 (20.5)	128.5 (21.31)	132.4 (19.3)	134.2 (19.9)	< 0.001	< 0.001
DBP (mm Hg), mean (SD)	80.5 (10.4)	81.4 (10.7)	80.3 (12.0)	81.1 (11.1)	80.8 (10.7)	0.891	0.932
Fasting plasma glucose (mmol/L), median (IQR)	7.8 (3.1)	8.1 (3.63)	8.2 (3.5)	8.2 (4.2)	7.6 (2.7)	0.029	0.376
Triglycerides (mmol/L), median (IQR)	1.8 (1.5)	1.8 (1.4)	1.9 (1.3)	1.9 (1.1)	1.6 (1.0)	0.012	0.004
Total cholesterol (mmol/L), median (IQR)	5.0 (1.3)	5.3 (1.6)	5.2 (1.2)	4.9 (1.3)	5.0 (1.5)	0.006	0.114
eGFR (ml/min per 1.73 m^2^), mean (SD)	83.6 (13.1)	74.6 (11.3)	69.4 (11.2)	66.6 (11.4)	61.6 (11.8)	< 0.001	0.114
Family history of CVD, (yes), n (%)	18 (9.1)	9 (4.6)	12 (6.1)	6 (3.0)	9 (4.6)	0.088	0.039
Hypertension, (yes), n (%)	129 (65.5)	139 (70.6)	141 (71.6)	157 (79.7)	160 (81.2)	0.001	< 0.001
Glucose lowering drugs (yes), n (%)	106 (53.8)	106 (53.8)	112 (56.9)	109 (55.3)	115 (58.4)	0.094	0.341
RAAS-I drugs, (yes), n (%)	17 (8.6)	22 (11.2)	40 (20.3)	32 (16.2)	47 (23.9)	< 0.001	< 0.001
History of laser treatment for diabetic retinopathy^†^, (yes), n (%)	14 (7.1)	22 (11.2)	22 (11.2)	25 (12.7)	21 (10.7)	0.464	0.213

^*^The characteristics were measured at the index date (Phase 4).

^†^Diabetic retinopathy defined as a positive history of severe non-proliferative and proliferative diabetic retinopathy during the exposure period.

**eGFR**: estimated glomerular filtration rate; **SD**: standard deviation; **BMI**: body mass index; **SBP**: systolic blood pressure; **DBP**: diastolic blood pressure;

**CVD**: cardiovascular disease; **IQR**: interquartile range; **RAAS-I**: renin-angiotensin-aldosterone system inhibitors

In individuals without diabetes (Table 2), significant differences were observed between quintiles in all characteristics. Moreover, moving from quintile 1 (representing steep decliners) to quintile 5 (indicating improvers) of the eGFR slope, there was a trend of elevated values for age, BMI, SBP, DBP, FPG, TG, TC, prevalence of hypertension, and use of RAAS-I drugs (all P values for trend < 0.001).

The characteristics of participants and non-participants at study enrollment are shown in Supplementary Table 4. Statistically significant differences in most characteristics were observed between the two groups; participants were younger and had lower mean SBP and DBP, but higher mean eGFR.

### Association between slopes and CVD

An overview of the HRs and 95% CI across quintiles of eGFR slope is provided in Tables 3 and 4 for individuals with and without diabetes, respectively. The slopes of eGFR on a continuous scale were not found to be associated with the risks of CVD in either individuals with or without diabetes. However, among individuals with diabetes (Table 3), we observed a negative linear association between the quintiles of eGFR slope and subsequent risk of CVD (P for linear trend < 0.05) despite adjustments for all study confounders (Model 3) including sex, age, BMI, smoking status, hypertension, hypercholesterolemia, FH-CVD, marital status, education level, FPG, glucose-lowering drugs, eGFR at index date, RAAS-I, and NPDR/PDR. In sex and age-adjusted models, individuals with diabetes in quintiles 2 and 4 had 83% and 61%, respectively, higher risk of CVD, compared to those in quintile 5. These associations remained significant in model 3, which included all proposed adjustments; subjects in quintile 2, had more than twice the risk of CVD (Hazard ratio (HR) 2.39 [95% CI 1.38–4.12]; P < 0.01) compared to those individuals in quintile 5. The corresponding value for quintile 4 was 1.76 (1.14–2.70; P < 0.01). Furthermore, in model 3, the association became significant for individuals in quintile 1 (2.16, 1.09–4.26; P < 0.05). Among individuals without diabetes (Table 4), no significant association was found between quintiles of eGFR slope and CVD events.

**Table 2:** Characteristics of participants without diabetes (n = 5934) according to quintiles of eGFR slopes at index date^*^

**Characteristics**	**Quintile 1** **(n = 1187)**	**Quintile 2** **(n = 1187)**	**Quintile 3** **(n = 1187)**	**Quintile 4** **(n = 1187)**	**Quintile 5** **(n = 1186)**	**P value**	**P trend**
Slope range (ml/min per 1.73 m^2^ per year)	(-1.15, -0.82)	(-0.82, -0.74)	(-0.74, -0.67)	(-0.67, -0.59)	(-0.59, -0.01)		
Slop, mean (SD)	-0.89 (0.06)	-0.78 (0.02)	-0.71 (0.02)	-0.63 (0.02)	-0.49 (0.08)	< 0.001	< 0.001
Age, mean (SD)	37.2 (7.0)	42.1 (8.7)	45.7 (9.5)	51.2 (10.5)	59.8 (11.5)	< 0.001	< 0.001
Female sex, n (%)	757 (63.8)	827 (69.7)	764 (64.4)	705 (59.4)	369 (31.1)	< 0.001	< 0.001
Educational level, n (%)							
< 6 years	83 (7.0)	145 (12.2)	195 (16.4)	323 (27.2)	457 (38.5)	< 0.001	< 0.001
6–12 years	762 (64.2)	714 (60.2)	714 (60.2)	647 (54.5)	526 (44.4)		< 0.001
> 12 years	342 (28.8)	328 (27.6)	278 (23.4)	217 (18.3)	203 (17.1)		< 0.001
Marital status, n (%)							
Single	152 (12.8)	100 (8.4)	85 (7.2)	50 (4.2)	30 (2.5)	< 0.001	< 0.001
Married	1006 (84.8)	1036 (87.3)	1033 (87.0)	1027 (86.5)	1008 (85.0)		0.931
Widowed/Divorced	29 (2.4)	51 (4.3)	69 (5.8)	110 (9.3)	148 (12.5)		< 0.001
Smoking status, n (%)							
Never smoker	864 (72.8)	941 (79.3)	900 (75.8)	891 (75.1)	779 (65.7)	< 0.001	< 0.001
Past smoker	58 (4.9)	58 (4.9)	73 (6.1)	90 (7.6)	178 (15.0)		< 0.001
Current smoker	265 (22.3)	188 (15.8)	214 (18.0)	206 (17.4)	2296 (19.3)		0.206
BMI (kg/m^2^), mean (SD)	27.2 (4.7)	27.9 (4.8)	28.7 (4.6)	29.0 (4.5)	28.0 (4.3)	< 0.001	< 0.001
SBP (mm Hg), mean (SD)	109.5 (13.6)	111.4 (14.6)	114.8 (15.5)	118.5 (17.2)	124.9 (19.8)	< 0.001	< 0.001
DBP (mm Hg), mean (SD)	74.5 (9.8)	75.7 (10.3)	77.7 (10.8)	78.5 (10.4)	79.6 (11.4)	< 0.001	< 0.001
Fasting plasma glucose (mmol/L), median (IQR)	5.0 (0.56)	5.1 (0.67)	5.2 (0.61)	5.2 (0.67)	5.3 (0.67)	< 0.001	< 0.001
Triglycerides (mmol/L), median (IQR)	1.2 (0.89)	1.2 (0.87)	1.4 (0.99)	1.5 (1.01)	1.5 (0.99)	< 0.001	< 0.001
Total cholesterol (mmol/L), median (IQR)	4.6 (1.1)	4.8 (1.1)	4.9 (1.2)	5.1 (1.3)	5.2 (1.3)	< 0.001	< 0.001
eGFR (ml/min per 1.73 m^2^), mean (SD)	91.5 (10.8)	83.7 (10.4)	79.2 (10.0)	73.6 (10.1)	66.9 (10.9)	< 0.001	< 0.001
Family history of CVD (yes), n (%)	63 (5.3)	72 (6.1)	64 (5.4)	80 (6.7)	46 (3.9)	0.034	0.296
Hypertension, (yes), n (%)	406 (34.2)	470 (39.6)	562 (47.3)	634 (53.4)	751 (63.3)	< 0.001	< 0.001
RAAS-I drugs, (yes), n (%)	8 (0.7)	21 (1.8)	24 (2.0)	53 (4.5)	74 (6.2)	< 0.001	< 0.001

^*^The characteristics were measured at the index date (Phase 4).

**eGFR**: estimated glomerular filtration rate; **SD**: standard deviation; **BMI**: body mass index; **SBP**: systolic blood pressure; **DBP**: diastolic blood pressure; **CVD**: cardiovascular disease; **IQR**: interquartile range; **RAAS-I**: renin-angiotensin-aldosterone system inhibitors

**Table 3:** Adjusted HRs for study outcomes according to the eGFR and its categories in subjects with diabetes (n = 985)

			Model 1		Model 2		Model 3		
	Events/n	Incidence rate per 1000 person-years (95% CI)	HR (95% CI)	P value	HR (95% CI)	P value	HR (95% CI)	P value	P trend*
**The slope of eGFR (**mL/min/1.73 m^2^ per year**)**			0.51 (0.12–2.11)	0.357	0.50 (0.11–2.18)	0.359	0.28 (0.05–1.42)	0.125	
**Quintiles of the slope of eGFR**									
Q5 (-0.5198, -0.1679)	50/197	37.8 (28.6–49.9)	Reference		Reference		Reference		0.010
Q1 (-1.0579, -0.7456)	22/197	14.5 (9.5–22.0)	1.63 (0.88–3.02)	0.118	1.65 (0.88–3.11)	0.116	2.16 (1.09–4.26)	0.026	
Q2 (-0.7454, -0.6711)	35/197	24.1 (17.3–33.5)	1.83 (1.10–3.05)	0.019	1.92 (1.15–3.22)	0.011	2.39 (1.38–4.12)	0.002	
Q3 (-0.6706, -0.6054)	36/197	24.8 (17.9–34.4)	1.39 (0.86–2.25)	0.176	1.36 (0.83–2.21)	0.211	1.54 (0.92–2.55)	0.094	
Q4 (-0.6053, -0.5203)	52/197	39.5 (30.1–51.8)	1.61 (1.07–2.43)	0.021	1.56 (1.03–2.37)	0.033	1.76 (1.14–2.70)	0.009	

Model 1 was adjusted for sex + age

Model 2 was adjusted for model 1 + BMI + smoking status + hypertension + hypercholesterolemia + FH-CVD + marital status + education level + FPG + glucose lowering drugs

Model 3 was adjusted for model 2 + eGFR at baseline (index date) + RAAS-I drugs + NPDR

* The p-value for the trend was computed in Model 3.

**HR**: hazard ratio; **eGFR**: estimated glomerular filtration rate; **CI**: confidence interval; **BMI**: body mass index; **FH-CVD**: family history of CVD; **FPG**: fasting blood glucose; **NPDR**: non-proliferative and proliferative diabetic retinopathy; **RAAS-I**: renin-angiotensin-aldosterone system inhibitors

**Table 4:** Adjusted HRs for study outcomes according to the eGFR and its categories in subjects without diabetes (n=5934)

			Model 1		Model 2		Model 3		
	Events/n	Incidence rate per 1000 person-years (95% CI)	HR (95% CI)	P value	HR (95% CI)	P value	HR (95% CI)	P value	P trend
**The slope of eGFR (**mL/min/1.73 m^2^ per year**)**			1.30 (0.49–3.42)	0.591	1.51 (0.56–4.03)	0.409	1.12 (0.37–3.39)	0.841	
**Quintiles of Slope of eGFR**									
Q5 (-0.5198, -0.1679)	167/1186	19.0 (16.3–22.1)	Reference		Reference		Reference		0.786
Q1 (-1.0579, -0.7456)	24/1187	2.5 (1.6–3.7)	0.87 (0.52–1.46)	0.619	0.96 (0.58–1.60)	0.899	1.09 (0.63–1.86)	0.755	
Q2 (-0.7454, -0.6711)	36/1187	3.8 (2.7–5.3)	0.91 (0.60–1.39)	0.686	0.98 (0.64–1.48)	0.925	1.06 (0.69–1.63)	0.789	
Q3 (-0.6706, -0.6054)	49/1187	5.2 (3.9–6.9)	0.90 (0.62–1.28)	0.569	0.89 (0.62–1.27)	0.527	0.96 (0.66–1.38)	0.826	
Q4 (-0.6053, -0.5203)	80/1187	8.5 (6.8–10.6)	1.01 (0.75–1.34)	0.962	0.96 (0.72–1.28)	0.806	0.99 (0.74–1.34)	0.988	

Model 1 was adjusted for sex + age

Model 2 was adjusted for model 1 + BMI + smoking status + hypertension + hypercholesterolemia + FH-CVD + marital status + education level + FPG

Model 3 was adjusted for model 2 + eGFR at baseline (index date) + RAAS-I drugs

* The p-value for the trend was computed in Model 3.

**HR**: hazard ratio; **eGFR**: estimated glomerular filtration rate; **CI**: confidence interval; **BMI**: body mass index; **FH-CVD**: family history of CVD; **FPG**: fasting blood glucose; **RAAS-I**: renin-angiotensin-aldosterone system inhibitors

**Fig. 2:**
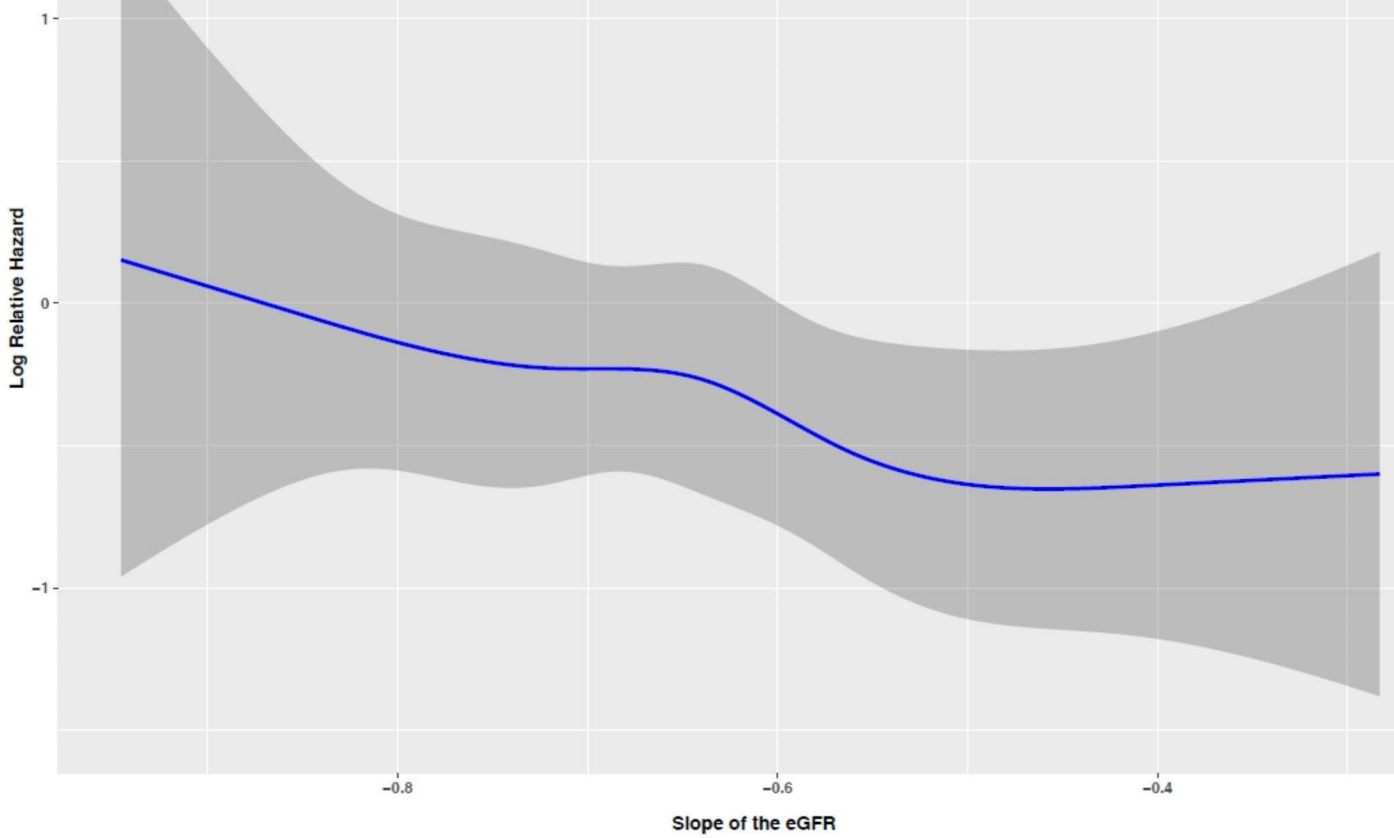
Spline curves showing log relative hazard (HRs) and 95%CIs (shaded) for CVD among individuals with diabetes. Knots were placed at the 5th, 25th, 50th, 75th, and 95th percentiles of the eGFR slope. The model was adjusted for the following variables (set at representative levels): sex (female), age (58 years), BMI (29.43 kg/m^2^), smoking status (never), hypertension (yes), hypercholesterolemia (yes), FH-CVD (no), marital status (married), education level (< 6 years), FPG (143 mg/dl), eGFR at index date (71.3) and glucose-lowering drugs (yes), RAAS-I drugs = no, NPDR = no. **HR**: hazard ratio; **eGFR**: estimated glomerular filtration rate; **CI**: confidence interval; **BMI**: body mass index; **FH-CVD**: family history of CVD; **FPG**: fasting blood glucose; **RAAS-I**: renin-angiotensin-aldosterone system inhibitors; **NPDR/PDR**: non-proliferative and proliferative diabetic retinopathy

**Fig. 3:**
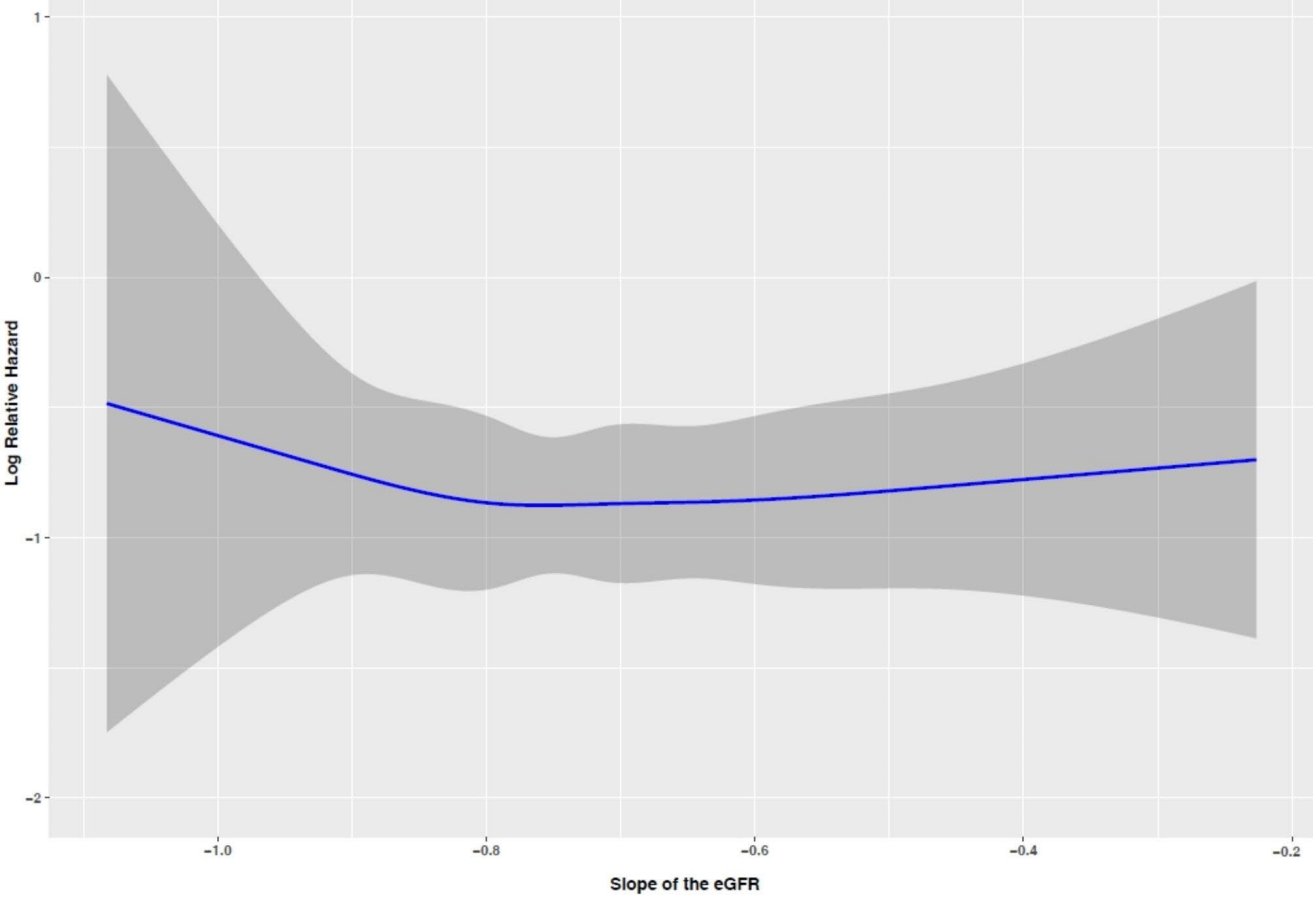
Spline curves showing Log HRs and 95%CIs (shaded) for CVD among individuals without diabetes Knots were placed at the 5th, 25th, 50th, 75th, and 95th percentiles of the eGFR slope. The model was adjusted for the following variables (set at representative levels): sex (female), age (45 years), BMI (27.7 kg/m^2^), smoking status (never), hypertension (no), hypercholesterolemia (no), FH-CVD (no), marital status (married), education level (6–12 years), FPG (93 mg/dl), eGFR at index date (78.8), RAAS-I drugs = no. **HR**: hazard ratio; **eGFR**: estimated glomerular filtration rate; **CI**: confidence interval; **BMI**: body mass index; **FH-CVD**: family history of CVD; **FPG**: fasting blood glucose; **RAAS-I**: renin-angiotensin-aldosterone system inhibitors

As shown in Figs. 2 and 3, our analysis found no evidence supporting a non-linear association between the eGFR slopes and CVD in individuals with and without diabetes, respectively (P for non-linearity: 0.726 and 0.837, respectively).

## Discussion

In this prospective, community-based cohort study, we examined the association between the slope of eGFR and the subsequent risk of CVD events in individuals with and without diabetes.

Our findings revealed that only among individuals with diabetes, a steeper decrease in eGFR over an exposure period of 9 years was associated with a higher risk of experiencing a cardiovascular event, independent of baseline kidney function, demographic factors, and traditional risk factors for CVD. Our results supported the utility of eGFR slopes for predicting CVD events in T2DM.

Multiple epidemiological studies have consistently demonstrated that individuals with chronic kidney disease (CKD) or those experiencing a decline in kidney function over time are at a higher risk of CVD events such as heart attacks, strokes, and heart failure, independent of traditional risk factors of CVD [6, 29]. Several mechanisms have been identified to explain these associations. As kidney function declines, waste products and toxins accumulate, triggering a chronic inflammation that contributes to the development and progression of CVD [29, 30]. Additionally, declining kidney function compromises blood pressure regulation, leading to hypertension, a major CVD risk factor [31]. Imbalances in minerals such as calcium and phosphate, which can occur with declining kidney function, result in vascular calcification and increased CVD risk [32]. Another mechanism is the activation of the Renin-Angiotensin-Aldosterone System (RAAS) in response to declining kidney function, promoting the production of angiotensin II and aldosterone, hormones that contribute to CVD [6].

It is widely recognized that people with diabetes have a more rapid decline in kidney function compared with those without diabetes [33]. Unexpectedly, our study revealed that individuals without diabetes experienced a higher average decline in eGFR, compared to those with diabetes. One potential explanation could be the phenomenon of hyperfiltration in patients with diabetes. Hyperfiltration refers to the kidneys filtering blood at a higher rate than normal, which is a compensatory mechanism in response to elevated glucose levels in the early stages of diabetes. However, prolonged hyperfiltration can eventually lead to kidney damage and the progression of diabetic kidney disease [34, 35]. Another possible explanation could be the effect of certain glucose-lowering drugs used in the treatment of diabetes, which may help slow down the decline in kidney function [36, 37]. However, the most prevalent glucose-lowering drugs used in the TLGS population [38] were sulfonylureas, either alone or in combination with metformin and/or insulin, and the use of drugs that have been shown to have a favorable impact on eGFR decline, such as SGLT2 inhibitors and GLP-1 analogs was very low. Another possible explanation for the observed discrepancy in our study could be the effect of baseline eGFR. There is some evidence to suggest that individuals with higher baseline eGFR tend to experience a more rapid decline in kidney function over time [39]. In our study, the individuals without diabetes had higher eGFR at the start of the study compared to the individuals with diabetes, which may contribute to the higher decline in eGFR observed in individuals without diabetes. Furthermore, it is well-known that dietary modifications, such as reducing salt and protein intake, can have a positive impact on kidney function [40, 41]. Therefore, the slower decline in eGFR observed in patients with diabetes may be attributed to their greater adherence to these dietary recommendations, compared to individuals without diabetes.

Only a few studies have investigated the relation between slopes of eGFR and the subsequent risk of CVD in various subgroups. In a cohort of 529,312 adults with a short follow-up time, from the Alberta Kidney Disease Network, it was found that an eGFR slope of -4 ml/min per 1.73 m^2^ per year was associated with a 74% higher risk of congestive heart failure, a 16% higher risk of acute MI, and a 21% higher risk of stroke, in comparison to individuals with no change in eGFR [42]. However, the mentioned study estimated the slope of eGFR using the least square method, which is known to have certain methodological limitations or defects [28].

In a cohort of individuals with T2DM in France [43], it was observed that those who experienced major CVD events had a greater annual decline in eGFR over a follow-up period of 6.3 years compared to those without CVD events (-3.0 vs. -1.7 ml/min per 1.73 m^2^ per year). However, this study primarily focused on individuals with T2DM, and the slope of eGFR was estimated for each patient using the least square method.

The ADVANCE-ON study, a clinical trial conducted in 20 countries, investigated the utility of the eGFR slope in predicting the risk of vascular outcomes and all-cause mortality in individuals with T2DM [13]. The findings showed that an annual substantial decrease in eGFR (according to quartiles of eGFR slope (<-1.63 ml/min per 1.73 m^2^ per year) was significantly associated with the 26% higher risk of the major CVD events, compared with no change in eGFR (0 ml/min per 1.73 m^2^ per year). However, this study focused on individuals with T2DM aged > 55 years and estimated slope for a short period time of 20 months.

Our study showed that individuals with diabetes who had a declining eGFR slope of -0.74 to -0.67 ml/min per 1.73 m^2^ per year, had more than twice the risk of experiencing CVD events compared to those with a slope in the highest quintile (Q5) (-0.51 to -0.16 ml/min per 1.73 m^2^ per year), even after adjusting for baseline eGFR. Despite the difference in the threshold for eGFR slope in our study compared to the above-mentioned studies [13, 42, 43], we observed similar associations between eGFR slope and CVD events among individuals with diabetes. The varying thresholds for eGFR slope across studies, influenced by differences in study populations, methodologies, and statistical approaches, did not diminish the consistent finding of an association between declining eGFR slope and increased CVD risk in individuals with diabetes. This reinforces the significance of monitoring kidney function and highlights the potential value of eGFR slope as a predictor of CVD in patients with diabetes.

Several previous studies have investigated the percentage and absolute annual change in eGFR, calculated from only two measurements of eGFR at baseline and follow-up, as predictors of CVD [44, 45]. To compare our findings with the mentioned studies, we computed the annual percentage change of eGFR using two specific points: the baseline phase and phase 4 (data not shown). The association between the annual percentage change of eGFR and CVD risk was examined on continuous scale, as well as by the categorization of study subjects into quartiles of annual eGFR percentage change (Q1-Q4), with Q4 as the reference group. Accordingly, among patients with diabetes, we found a non-significantly increased risk of CVD in Q1 (HR: 1.21, CI: 0.77–1.90) and Q2 (1.23; 0.80–1.88) compared with Q4, after adjusting for all study covariates. This finding highlights that eGFR changes assessed by slope analyses using LMMs are a more effective tool for capturing the risk of CVD in patients with diabetes than the percentage change of eGFR.

In our study, no significant association was found between the eGFR slope and CVD in individuals without diabetes. The lack of available data on the population without diabetes limits our ability to make direct comparisons. However, a study that used the least-square method to estimate the eGFR slope found that a slope of < -3 ml/min per 1.73 m^2^ per year over 5 years predicted CVD events in the general population, independent of the baseline eGFR [46]. The discrepancy between our findings and this study could be attributed to several factors, including the range of eGFR slope and the threshold used to define a significant decline in eGFR. In our study, the range of eGFR decline in subjects without diabetes was − 1.15 to -0.01, whereas, in the mentioned study, it was − 5 to 1.00. Therefore, the relatively modest decrease in eGFR observed in our study may not pose a significant CVD risk for individuals without diabetes.

While certain clinical guidelines have proposed the use of eGFR slope as a surrogate endpoint for predicting ESKD and have defined a threshold for it [12], there is still no clear cutoff established for eGFR slope in relation to CVD risk. Therefore, further research and consensus among experts are necessary to establish a clear definition and standardized criteria for considering eGFR slope as a predictor or surrogate endpoint in clinical trial designs and decision-making processes related to CVD management and treatment.

Our study has several strengths. First, we included a large population encompassing individuals both with and without diabetes, enabling us to examine the relationships separately within each group. Secondly, our study had a substantial duration for both the ascertainment of the eGFR slope and the follow-up period for outcome evaluation. Furthermore, our study included sequential measurements of eGFR during the exposure period. This approach enabled us to capture the dynamic nature of kidney function and its relationship to the outcomes being investigated. Moreover, we utilized LMEM to estimate eGFR slopes which are known for their reliability and are particularly suitable for analyzing data with varying intervals between measurements and a highly heterogeneous number of eGFR [28]. Many previous studies have relied on individual linear regression to estimate the slope of eGFR [46, 47]. However, this method fails to adequately account for the varying number of eGFR measurements, leading to potential inaccuracies. In contrast, the LMEM incorporates partial pooling, giving more weight to participants with more observations (i.e., eGFR measures). This approach enhances the precision of the estimates, particularly when some individuals have a limited number of measurements [28].

Our study is limited in that we used eGFR instead of a directly measured GFR to calculate GFR slopes, which may lead to some misclassification of the true course of change in kidney function [48, 49]. Second, we did not have data on albuminuria. However, a large-scale meta-analysis [9] and a recent cohort study [50] have suggested that eGFR decline itself is a robust determinant of several outcomes (i.e., ESRD and mortality events), independent of albuminuria. Furthermore, according to a study in 2020, researchers concluded that both early changes in albuminuria and the slope of GFR fulfilled the criteria for surrogacy for use as an endpoint in clinical trials for CKD progression, with stronger support for change in GFR than albuminuria [51]. Thirdly, in our study, the number of individuals with diabetes was lower compared to those without diabetes. However, the results of the power calculation indicated that our study had a significant statistical power ranging from 80 to 99% to detect an HR of 1.54 and 2.16 for incident CVD in individuals with diabetes. Fourthly, as a result of the limited number of CVD outcomes, we faced limitations in stratifying the analysis for all individual components of the composite CVD. Lastly, the associations we observed within our study population may not necessarily be generalizable to other populations with different demographic, clinical, or genetic characteristics.

## Conclusion

In conclusion, our study provides valuable insights into the association between eGFR slope and CVD in individuals with and without diabetes. We found that a steeper decline in the eGFR slope was significantly associated with a higher risk of CVD events in individuals with diabetes, even after adjusting for baseline eGFR. However, we did not observe a significant association between the eGFR slope and CVD in individuals without diabetes. These findings highlight the importance of monitoring kidney function and considering the eGFR slope as a potential predictor of CVD risk in individuals with diabetes.

### Electronic supplementary material

Below is the link to the electronic supplementary material.

Supplementary Material 1

## Data Availability

All datasets generated and analyzed during the current study are available from the corresponding author upon reasonable request.

## List of Abbreviations

eGFR: Estimated glomerular filtration rate
CVD: Cardiovascular disease
TLGS: Tehran Lipid and Glucose Study
MENA: Middle East and North Africa
CKD: Chronic kidney disease
ESRD: End-stage renal disease
FH-CVD: Family history of CVD
BMI: Body mass index
SBP: Systolic blood pressure
DBP: Diastolic blood pressure
FPG: Fasting plasma glucose
2-hPG: 2-h post-load plasma glucose
TC: Total cholesterol
TG: Triglycerides
HDL-C: High-density lipoprotein cholesterol
CKD-EPI: Chronic Kidney Disease Epidemiology Collaboration
Scr: Serum creatinine
T2DM: Type 2 diabetes
MI: Myocardial infarction
ECG: Electrocardiogram
CHF: Congestive heart failure
LMEM: Linear mixed-effects models
IQR: Interquartile range
CI: Confidence interval
ANOVA: Analysis of variance
RAAS: Renin-angiotensin-aldosterone system
NPDR: Non-proliferative diabetic retinopathy
PDR: Proliferative diabetic retinopathy
