# Supplementary material for: Association between estimated glomerular filtration rate slope and cardiovascular disease among individuals with and without diabetes: a prospective cohort study
Source: Cardiovasc Diabetol. 2023 Oct 4;22:270. doi: 10.1186/s12933-023-02008-x (PMC10552420; doi:10.1186/s12933-023-02008-x)
Supplement: Supplementary file 1 — Supplementary Material 1 [file 12933_2023_2008_MOESM1_ESM.docx]

| **Supplementary Table 1:** The number of eGFR measures categorized by sex, diabetes status, and quintile of slopes | | | | | |
| --- | --- | --- | --- | --- | --- |
|  | | | **Two measures^*^**  **n (%)** | **Three measures**  **n (%)** | **Four measures**  **n (%)** |
| **Men** (n=2899) | | | 341 (11.7) | 1063 (36.7) | 1495 (51.6) |
| **Women** (n=4020) | | | 327 (8.1) | 1288 (32.0) | 2405 (59.8) |
|  | | |  |  |  |
| **Population with diabetes** (n=985) | | | 53 (5.4) | 304 (30.9) | 628 (63.8) |
| **Population without diabetes** (n=5934) | | | 616 (10.4) | 2047 (34.5) | 3271 (55.1) |
|  | | |  |  |  |
| **Quintile of eGFR slope** | | |  |  |  |
| **Population with diabetes** | Quintile 1 (n=197) | | 10 (18.9) | 62 (20.4) | 125 (19.9) |
|  | Quintile 2 (n=197) | | 10 (18.9) | 57 (18.8) | 130 (20.7) |
|  | Quintile 3 (n=197) | | 13 (24.5) | 61 (20.1) | 123 (19.6) |
|  | Quintile 4 (n=197) | | 12 (22.6) | 69 (22.7) | 116 (18.5) |
|  | Quintile 5 (n=197) | | 8 (15.1) | 55 (18.1) | 134 (21.3) |
|  | | |  |  |  |
| **Population without diabetes** | | Quintile 1 (n=1187) | 150 (24.4) | 413 (20.2) | 624 (19.1) |
|  |  | Quintile 2 (n=1187) | 137 (22.2) | 424 (20.7) | 626 (19.1) |
|  |  | Quintile 3 (n=1187) | 125 (20.3) | 421 (20.6) | 641 (19.6) |
|  |  | Quintile 4 (n=1187) | 106 (17.2) | 419 (20.5) | 662 (20.2) |
|  |  | Quintile 5 (n=1186) | 98 (15.9) | 370 (18.1) | 718 (22.0) |
| Number of eGFR measures during exposure period; **eGFR**: estimated glomerular filtration rate | | | | | |

| **Supplementary Table 2:** Characteristics of participants, overall and stratified by diabetes status at the time of study enrollment^*^ | | | | |
| --- | --- | --- | --- | --- |
| **Characteristics** | **Total population**  **(n=6919)** | **With diabetes**  **(n=985)** | **Without diabetes**  **(n=5934)** | **P Value** |
| Age, mean (SD) | 39.5 (12.6) | 48.2 (11.1) | 38.0 (12.2) | <0.001 |
| Female sex, n (%) | 4020 (58.1) | 597 (60.6) | 3423 (57.7) | 0.083 |
| Educational level, n (%) |  |  |  |  |
| < 6 years | 1756 (25.4) | 462 (47.0) | 1294 (21.8) | <0.001 |
| 6-12 years | 4152 (60.1) | 433 (44.0) | 3719 (62.7) |  |
| > 12 years | 1003 (14.5) | 88 (9.0) | 915 (15.4) |  |
| Marital status, n (%) |  |  |  |  |
| Single | 1051 (15.2) | 34 (3.5) | 1017 (17.1) | <0.001 |
| Married | 5590 (80.8) | 866 (87.9) | 4724 (79.6) |  |
| Widowed/Divorced | 277 (4.0) | 85 (8.6) | 192 (3.2) |  |
| Smoking status, n (%) |  |  |  |  |
| Never smok­er | 5425 (79.5) | 762 (78.4) | 4663 (79.6) | 0.001 |
| Past smoker | 424 (6.2) | 85 (8.7) | 339 (5.8) |  |
| Current smoker | 978 (14.3) | 125 (12.9) | 853 (14.6) |  |
| BMI (kg/m^2^), mean (SD) | 26.6 (4.6) | 29.4 (4.5) | 26.1 (4.4) | <0.001 |
| SBP (mm Hg), mean (SD) | 116.1 (16.5) | 126.5 (19.1) | 114.4 (15.3) | <0.001 |
| DBP (mm Hg), mean (SD) | 76.3 (10.3) | 81.6 (10.4) | 75.5 (10.0) | <0.001 |
| Fasting plasma glucose (mmol/L), median (IQR) | 4.9 (0.6) | 6.1 (2.3) | 4.8 (0.6) | <0.001 |
| Triglycerides (mmol/L), median (IQR) | 1.5 (1.2) | 2.2 (1.5) | 1.4 (1.1) | <0.001 |
| Total cholesterol (mmol/L), median (IQR) | 5.1 (1.5) | 5.6 (1.6) | 5.0 (1.4) | <0.001 |
| eGFR (ml/min per 1.73 m^2^), mean (SD) | 82.9 (14.7) | 76.1 (13.8) | 83.9 (14.6) | <0.001 |
| Hypertension, n (%) | 2855 (41.8) | 642 (66.0) | 2213 (37.8) | <0.001 |
| Glucose lowering drugs, n (%) | 165 (2.4) | 165 (16.8) | - | - |
| RAAS-I drugs, (yes), n (%) | 66 (1.0) | 29 (2.9) | 37 (0.6) | <0.001 |
| *The characteristics were measured upon enrollment to the study (Phase 1 or 2).  **eGFR:** estimated glomerular filtration rate; **SD:** standard deviation; **BMI:** body mass index; **SBP:** systolic blood pressure; **DBP:** diastolic blood pressure; **CVD:** cardiovascular disease; **IQR:** interquartile range; **RAAS-I:** renin-angiotensin-aldosterone system inhibitors | | | | |

| **Supplementary Table 3:** Characteristics of participants, overall and stratified by diabetes status at index date^*^ | | | | |
| --- | --- | --- | --- | --- |
| **Characteristics** | **Total population**  **(n=6919)** | **With diabetes**  **(n=985)** | **Without diabetes**  **(n=5934)** | **P Value** |
| Slope (ml/min per 1.73 m^2^ per year), mean (SD) | -0.69 (0.14) | -0.63 (0.13) | -0.70 (0.14) | <0.001 |
| Age, mean (SD) | 48.6 (12.7) | 57.5 (11.0) | 47.2 (12.3) | <0.001 |
| Female sex, n (%) | 4020 (58.1) | 597 (60.6) | 3422 (57.7) | 0.083 |
| Educational level, n (%) |  |  |  |  |
| < 6 years | 1661 (24.0) | 458 (46.5) | 1203 (20.3) | <0.001 |
| 6-12 years | 3789 (54.8) | 426 (43.2) | 3363 (56.7) |  |
| > 12 years | 1469 (21.2) | 101 (10.3) | 1368 (23.1) |  |
| Marital status, n (%) |  |  |  |  |
| Single | 432 (6.2) | 15 (1.5) | 417 (7.0) | <0.001 |
| Married | 5918 (85.5) | 808 (82.0) | 5110 (86.1) |  |
| Widowed/Divorced | 569 (8.2) | 162 (16.4) | 407 (6.9) |  |
| Smoking status, n (%) |  |  |  |  |
| Never smok­er | 5130 (74.1) | 755 (76.6) | 4375 (73.7) | <0.001 |
| Past smoker | 553 (8.0) | 96 (9.7) | 457 (7.7) |  |
| Current smoker | 1236 (17.9) | 134 (13.6) | 1102 (18.6) |  |
| BMI (kg/m^2^), mean (SD) | 28.5 (4.8) | 30.2 (5.3) | 28.2 (4.6) | <0.001 |
| SBP (mm Hg), mean (SD) | 117.6 (18.2) | 128.3 (20.5) | 115.8 (17.2) | <0.001 |
| DBP (mm Hg), mean (SD) | 77.7 (10.8) | 80.3 (11.0) | 77.2 (10.7) | <0.001 |
| Fasting plasma glucose (mmol/L), median (IQR) | 5.2 (0.7) | 7.9 (3.5) | 5.1 (0.6) | <0.001 |
| Triglycerides (mmol/L), median (IQR) | 1.5 (1.0) | 1.8 (1.3) | 1.4 (0.9) | <0.001 |
| Total cholesterol (mmol/L), median (IQR) | 4.9 (1.3) | 5.1 (1.4) | 4.9 (1.2) | <0.001 |
| eGFR (ml/min per 1.73 m^2^), mean (SD) | 77.9 (13.8) | 71.2 (14.0) | 79.0 (13.4) | <0.001 |
| Family history of CVD, n (%) | 379 (5.5) | 54 (5.5) | 325 (5.5) | 0.995 |
| Hypertension, n (%) | 3549 (51.3) | 726 (73.7) | 2823 (47.6) | <0.001 |
| Glucose lowering drugs, n (%) | 548 (7.9) | 548 (55.6) | - | - |
| RAAS-I drugs, (yes), n (%) | 338 (4.9) | 158 (16.0) | 180 (3.0) | <0.001 |
| *The characteristics were measured at the index date (Phase 4).  **eGFR:** estimated glomerular filtration rate; **SD:** standard deviation; **BMI:** body mass index; **SBP:** systolic blood pressure; **DBP:** diastolic blood pressure; **CVD:** cardiovascular disease; **IQR:** interquartile range; **RAAS-I:** renin-angiotensin-aldosterone system inhibitors | | | | |

| **Supplementary Table 4:** Baseline characteristics of participants and non-participants | | | |
| --- | --- | --- | --- |
| ***Characteristics** | **Participants**  **(n=6919)** | **Non-participants**  **(n=4771)** | **P Value** |
| Age, mean (SD) | 39.5 (12.6) | 41.0 (14.7) | <0.001 |
| Female sex, n (%) | 4020 (58.1) | 2680 (56.2) | 0.038 |
| Educational level, n (%) |  |  |  |
| < 6 years | 1756 (25.4) | 1478 (31.0) | <0.001 |
| 6-12 years | 4152 (60.1) | 2642 (55.5) |  |
| > 12 years | 1003 (14.5) | 643 (13.5) |  |
| Marital status, n (%) |  |  |  |
| Single | 1051 (15.2) | 863 (18.1) | <0.001 |
| Married | 5590 (80.8) | 3624 (76.0) |  |
| Widowed/Divorced | 277 (4.0) | 284 (6.0) |  |
| Smoking status, n (%) |  |  |  |
| Never smok­er | 5425 (79.5) | 3370 (73.4) | <0.001 |
| Past smoker | 424 (6.2) | 307 (6.7) |  |
| Current smoker | 978 (14.3) | 917 (20.0) |  |
| BMI (kg/m^2^), mean (SD) | 26.6 (4.6) | 26.7 (5.1) | 0.191 |
| SBP (mm Hg), mean (SD) | 116.1 (16.5) | 119.1 (20.0) | <0.001 |
| DBP (mm Hg), mean (SD) | 76.3 (10.3) | 77.4 (11.3) | <0.001 |
| Fasting plasma glucose (mmol/L), median (IQR) | 4.9 (0.6) | 4.9 (0.8) | <0.001 |
| Triglycerides (mmol/L), median (IQR) | 1.5 (1.2) | 1.5 (1.3) | 0.063 |
| Total cholesterol (mmol/L), median (IQR) | 5.1 (1.5) | 5.1 (1.6) | 0.438 |
| eGFR (ml/min per 1.73 m^2^), mean (SD) | 82.8 (14.7) | 81.4 (16.4) | <0.001 |
| Family history of CVD, n (%) | 1022 (14.8) | 767 (16.1) | 0.054 |
| Hypertension, n (%) | 2855 (41.8) | 2134 (46.7) | <0.001 |
| Glucose lowering drugs, n (%) | 165 (2.4) | 246 (5.2) | <0.001 |
| RAAS-I drugs, (yes), n (%) | 66 (1.0) | 101 (2.1) | <0.001 |
| *****Characteristics were measured at the start of the study (phase 1/phase 2)  **eGFR:** estimated glomerular filtration rate; **SD:** standard deviation; **BMI:** body mass index; **SBP:** systolic blood pressure; **DBP:** diastolic blood pressure; **CVD:** cardiovascular disease; **IQR:** interquartile range; **RAAS-I:** renin-angiotensin-aldosterone system inhibitors | | | |

| 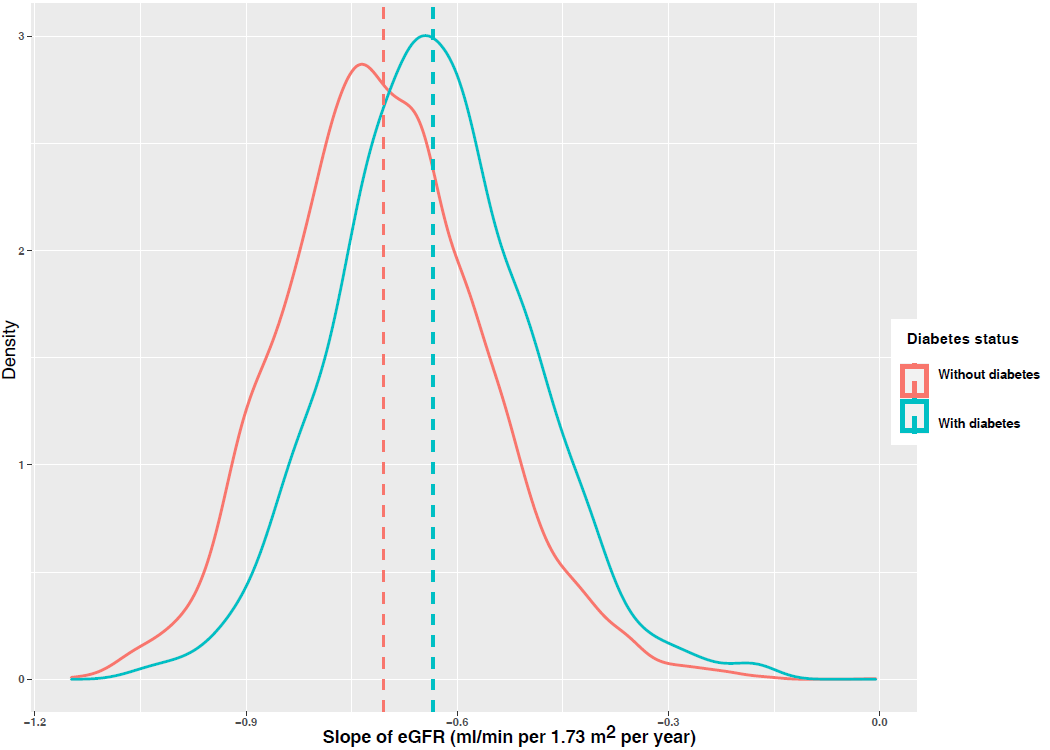 |
| --- |
| **Supplementary Figure 1:** Distribution of annual eGFR slopes over a median follow-up of 9 years, by diabetes status; **eGFR**: estimated glomerular filtration rate |

| 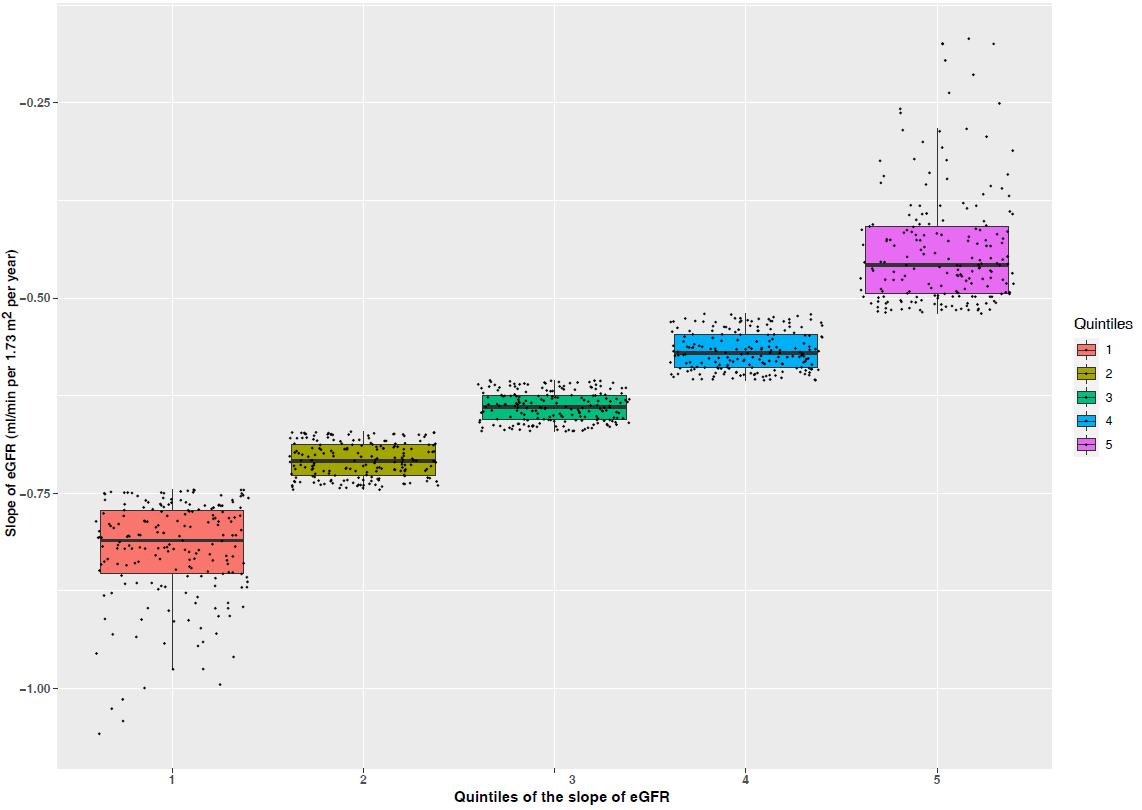 |
| --- |
| **Supplementary Figure 2:** Distribution of annual eGFR slopes among individuals with diabetes across quintiles of slopes; **eGFR**: estimated glomerular filtration rate |

| 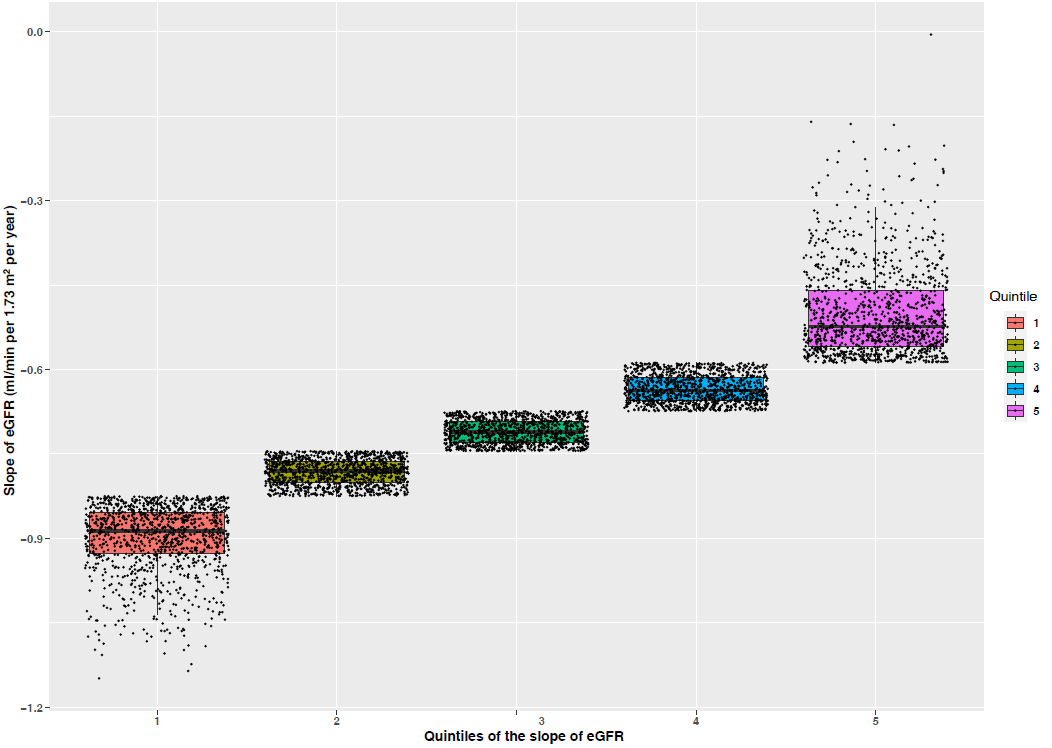 |
| --- |
| **Supplementary Figure 3:** Distribution of annual eGFR slopes among individuals without diabetes across quintiles of slopes; **eGFR**: estimated glomerular filtration rate |
